# RAS variant signalling

**DOI:** 10.1042/BST20180173

**Published:** 2018-10-04

**Authors:** Stephanie P. Mo, Judy M. Coulson, Ian A. Prior

**Affiliations:** Division of Cellular and Molecular Physiology, Institute of Translational Medicine, University of Liverpool, Liverpool L69 3BX, U.K.

**Keywords:** cancer, codon, isoforms, mutation, RAS, signalling

## Abstract

RAS proteins are small GTPases that regulate signalling networks that control cellular proliferation and survival. They are frequently mutated in cancer and a commonly occurring group of developmental disorders called RASopathies. We discuss recent findings describing how RAS isoforms and different activating mutations differentially contribute to normal and disease-associated biology and the mechanisms that have been proposed to underpin this.

## Introduction

**Figure 1.**
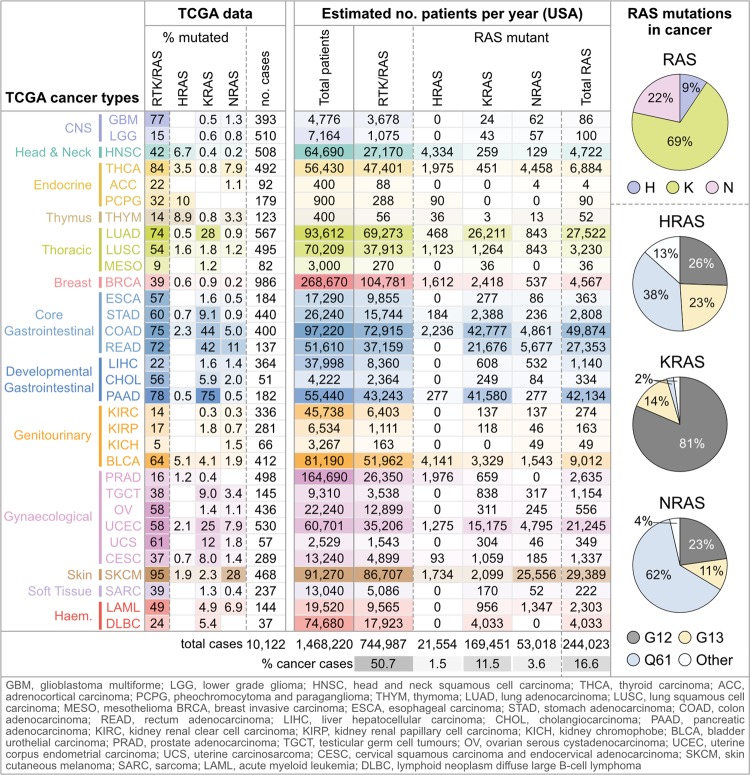
RAS mutations in cancer. RAS mutation frequencies for each cancer type are derived from TCGA PanCancer Atlas dataset, release 12.0, comprising 10 122 patient cases. RTK/RAS pathway mutation frequencies comprising data from 85 curated RAS pathway nodes are derived from Sanchez-Vega et al. [2]. Estimated annual RAS mutant patients in the U.S.A. calculated by multiplying the relevant RAS mutation frequencies by the estimated number of new patients per year per cancer type collated from American Cancer Society [63]. Codon mutation pie charts indicate the % of all RAS mutant cases and were collated from the COSMIC database, v85, comprising 53 728 RAS mutant samples [64].

RAS proteins are small GTPases that act as molecular switches controlling signalling pathways regulating cell proliferation and cell survival. When bound to guanosine triphosphate (GTP), RAS becomes active and signals via networks that include RAF/MEK/ERK and PI3K/AKT. Dysregulated RAS signalling is associated with oncogenesis and developmental disorders called RASopathies [1]. Approximately 17% of cancer patients harbour an activating RAS mutation (Figure 1) and oncogenic mutations within the wider receptor tyrosine kinase (RTK)–RAS network are found in ∼50% of all cancer patients [2,3]. Similarly, RASopathies represent a frequently occurring group of developmental disorders with at least 1 in 1000 patients harbouring mutations in RAS isoforms or RAS pathway components [4]. Almost all oncogenic mutations occur at codons G12, G13 and Q61 resulting in constitutively active RAS signalling [3,5]. These oncogenic mutations are typically embryonic lethal [6], whereas RASopathy-associated mutations tend to occur at other codons where they have milder RAS-activating effects [7].

**Figure 2.**
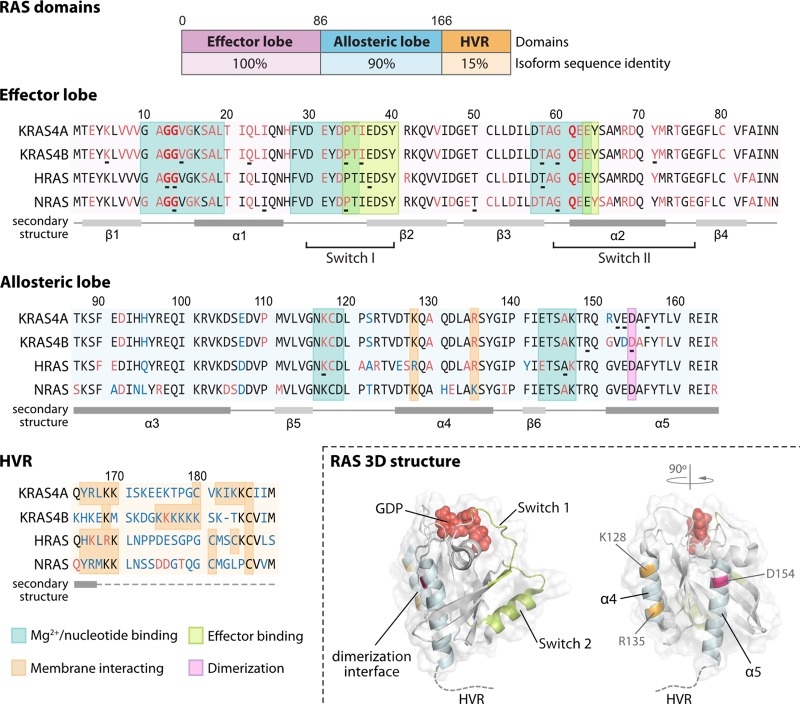
Sequence features of RAS isoforms. The G-domain of RAS comprises the effector and allosteric lobes. The main area of sequence divergence (blue) between isoforms is in the HVR with limited divergence also in the allosteric lobe clustered around residues associated with nucleotide and membrane binding. Residues mutated in cancer (red) and RASopathies (underlined) are highlighted. Wild-type KRAS 3D structure (PDB: 4OBE; residues 1–169) showing relative positioning of Switch domains versus membrane-binding residues regulating G-domain orientation (orange), D154 residue required for dimerisation (pink) and putative dimerisation interface on α4–α5 helices (cyan).

In humans, there are four ubiquitously expressed RAS isoforms encoded by three genes: *HRAS, NRAS* and *KRAS*, the transcript from the latter can be alternatively spliced into KRAS4A and KRAS4B. They share almost complete sequence homology (90%) in their G-domain (Figure 2). The G-domain (residues 1–166) is composed of an effector lobe (residues 1–86) and an allosteric lobe (87–166). The effector lobe is essential for interactions with RAS effectors such as RAF, PI3K and RalGEF, whereas the allosteric lobe plays a role in intra-protein communication where it connects the active site of the effector lobe to membrane-interacting residues [8]. When RAS is activated, conformational changes occur in the switch I (aa 30–40) and switch II (aa 60–76) regions to allow for effector interactions and signalling (Figure 2). The main difference between the isoforms is found at the carboxyl-terminal hypervariable region (HVR). This region is responsible for the membrane tethering of RAS proteins that is required for signalling [9]. Ras isoforms have alternative post-translational modifications of their HVR, which result in differences in membrane trafficking and localisation that are thought to contribute to isoform-specific signalling [10].

## Isoform-specific RAS signalling

### Ras isoform contributions to disease

Despite their sequence similarity, RAS proteins are not functionally redundant. Mouse development studies point to subtly different requirements for RAS isoforms: KRAS4B was thought to be the only isoform that was essential for normal development and growth in mice [11–13]. However, gene swap experiments where HRAS was inserted into the KRAS locus generated mice that survived to adulthood, albeit with evidence of cardiomyopathy [14]. Therefore, while it appears evident that generic RAS signalling is essential for normal development of most tissues, it seems that the spatiotemporal pattern of KRAS expression rather than unique signalling pathways associated with this isoform is the most critical feature for development. In fact, KRAS exhibits the highest abundance of RAS isoform mRNA transcripts in all tissues and KRAS4A is the most dynamically regulated isoform during embryogenesis [15]. KRAS4A expression is specifically up-regulated in embryonic heart during the phase when cardiac myocytes exit the cell cycle and undergo hypertrophy, suggesting that KRAS4A could play an important role in cardiovascular development [15].

Further evidence for subtle isoform-specific contributions to development are seen with germline mutations of RAS isoforms in humans that lead to developmental issues associated with chronic up-regulation of the RAS–RAF–MEK–ERK pathway. These RASopathies display a range of overlapping phenotypes frequently characterised by dysmorphic craniofacial features, short stature and learning disabilities [4,16]. Each RAS isoform is associated with a distinct subset of syndromes within the RASopathy group, indicating that there are observable differences in the developmental consequences of harbouring activating mutations in each isoform. For example, only KRAS mutations have been found in cardiofaciocutaneous syndrome, characterised by thick scaly skin and cardiac malformations, whereas mutations in HRAS mutant Costello syndrome are associated with higher risk of rhabdomyosarcomas and neuroblastomas [17]. These phenotypic differences highlight the developmental consequences of activating mutations in different Ras isoforms.

RAS isoforms have different mutation frequencies and display bias towards certain cancers. An anomaly in the field is the discrepancies between values often quoted for RAS mutations ranging from 15 to 30% of all cancers harbouring a RAS mutation and 60 to 98% of pancreatic cancers harbouring KRAS mutations [3,5]. This is partly due to different data sources and the sampling bias within datasets that do not reflect the relative population incidence of cancer types and/or the lack of comprehensive screening for mutations in all isoforms. The TCGA PanCancer Atlas represents a highly curated dataset of 10 122 cases where the disease type is verified and all samples have been comprehensively molecularly profiled including genome-wide screening for mutations [18]. Applying the TCGA-derived % incidence of RAS mutations for each TCGA cancer type to current American Cancer Society data on cancer rates reveals that ∼250 000 new cancer cases per year in the U.S.A. (17% of total) will harbour a RAS mutation (Figure 1). KRAS is the most frequently mutated RAS isoform (69% of RAS mutant patients) and is predominantly associated with pancreatic, lung and colorectal carcinoma (Figure 1). Notably, this analysis suggests that 75% of pancreatic cancer cases are KRAS mutated rather than the widely quoted >90%.

NRAS (22%) and HRAS (9%) isoforms are less frequently mutated with melanomas accounting for half of NRAS mutant patients, while head and neck cancer and bladder cancer together account for half of HRAS mutant patients. Given that farnesyltransferase inhibitors (FTIs) only target HRAS effectively [19], FTIs have the potential to benefit up to 21 500 new HRAS mutated patients per year in the U.S.A. While head and neck, thyroid or bladder cancers represent obvious targets for FTI clinical trials, the estimated patient numbers make a case for wider screening, including cancers not normally thought to be associated with HRAS such as colon (∼2236 new HRAS mutant patients per year), melanoma (∼1734 patients) and breast cancer (∼1612 patients).

The mechanistic basis for differences in RAS isoform mutation frequencies is unclear. Highly cited *in vitro* studies have shown that RAS isoforms appear to be differentially coupled to key effector pathways. Specifically, KRAS is a more efficient activator of RAF1 and RAC than other RAS isoforms [20,21], whereas HRAS favours PI3K activation more than KRAS [22]. However, this seems unlikely to be generic given the context-dependence of cell signalling. Indeed, recent large-scale studies measuring endogenous mutant KRAS signalling dependencies across a wider network of RAS signalling nodes showed significant heterogeneity in effector requirements between cell lines [23,24]. Although this might suggest that isoform-specific differences may be hard to discern in this noisy background, *in vivo* work demonstrates that functional differences exist between RAS isoforms. Genetically engineered mice inducibly expressing KRAS^G12D^ and NRAS^G12D^ from their endogenous loci exhibited clear phenotypic differences [25]. In the colonic epithelium, activated KRAS promoted hyperplasia by an MEK-dependent increase in the number of proliferative progenitor cells; in contrast, mutant NRAS conferred resistance to apoptosis. *In vitro* studies have shown that mutant KRAS can specifically promote the proliferation of undifferentiated endodermal stem cells that seed colon cancer, while mutant HRAS instead promoted growth arrest and differentiation into endoderm [26]. Taken together, these phenotypic differences could be a contributing factor to the predominance of KRAS mutations in cancers from endodermally derived tissues such as pancreas, lung and colon [27].

More recently, another mechanism based on relative expression levels has been proposed to explain why KRAS is more oncogenic than the other RAS isoforms. In mice, the *KRAS* gene exhibits a bias towards rare codons compared with the other RAS isoforms [28]. Rare codons can hinder protein translation [29], and alteration of rare codons to common codons was able to increase endogenous protein expression of KRAS [28]. Since high RAS expression can lead to senescence and oncogenic stress [30], it was proposed that the poor expression of KRAS protein caused by rare codons might avoid these outcomes and therefore give a higher tumourigenic potential. Consistent with this, a mouse model with codon optimised KRAS genes generated fewer urethane-induced tumours than unaltered controls [31]. While it is clear that RAS expression levels can affect on oncogenesis, it is also important to note that RAS isoform protein expression levels were not directly measured. Therefore, the key prediction that KRAS protein is expressed at lower levels than the other isoforms was not formally demonstrated. A challenge to the model also comes from other studies that have compared RAS protein levels and observed that KRAS is frequently expressed at higher protein levels than the other isoforms [32,33].

### Structural features underpinning isoform-specific RAS biology

Beyond potential differences in expression, attention has focussed on structural features of RAS isoforms to explain differences in their signalling capacity. Although the G-domain is highly conserved between RAS isoforms, recent research has implicated the allosteric lobe in mediating isoform-specific effects on their structure and biochemistry. Most of the residue differences between isoforms in the allosteric lobe occur at sites mediating RAS–membrane and RAS–nucleotide interactions (Figure 2) [8]. These residues are thought to influence the measured difference in GTP hydrolysis rates between the three isoforms and the propensity of Ras isoforms to occupy signalling-competent/incompetent conformational states [34]. In the GTP-bound active state, RAS samples two different conformations: state 1 (‘open’) that is less capable of binding effectors and more susceptible to nucleotide exchange and state 2 (‘closed’) that favours effector interactions and GTP hydrolysis [35]. Molecular dynamics and NMR analysis revealed that KRAS is inherently more flexible than the other isoforms and spends more time in the ‘open’ state, while HRAS and NRAS are predominantly in the ‘closed’ state [36,37]. In contrast with wild-type KRAS, KRAS^G12D^ favours the closed state, indicating how oncogenic mutations can shift the balance towards a more signalling-competent state by promoting stable effector interaction conformations while simultaneously inhibiting GTP hydrolysis to stabilise the GTP-bound state [37].

The allosteric lobe also transmits nucleotide-dependent changes in the effector lobe to specify how the G-domain and HVR interact and orientate with the membrane. Basic residues in the allosteric lobe electrostatically interact with plasma membrane phospholipids. When HRAS is GDP-bound this is mediated by R169 and K170, and when GTP binding occurs this switches to R128 and R135 [38]. The effect of swapping between these two sets of residues is to re-orientate the G-domain with respect to the membrane and to facilitate effector engagement [38]. NRAS and KRAS membrane orientations are more influenced by the HVR resulting in a spectrum of orientations that are preferentially adopted by distinct RAS isoforms that affect on their availability for effector engagement [39,40]. An important note is that these charged residues lie in the α4–α5 helices also thought to mediate RAS dimerisation (discussed later; Figure 2), and the potentially competitive interplay between dimerisation and distinct membrane interactions has not yet been explored.

### Ras interactions with the plasma membrane

As the primary mediator of membrane attachment, the HVR has a significant influence on isoform-specific RAS behaviour. All RAS isoforms are C-terminally farnesylated to provide weak membrane binding that is enhanced by a second signal comprising either palmitoylation for HRAS, NRAS and KRAS4A and/or a stretch of polylysine residues for KRAS4A and KRAS4B (Figure 2) [10]. The transient and dynamic nature of these modes of membrane interaction specifies different partitioning of each RAS isoform between endomembranes and the cell surface. RAS nanoclusters are small (10 nm radius, 6–7 RAS proteins), transient (<1 s) platforms that act as digital relays within RAS signalling circuits [41]. Each RAS isoform occupies distinct non-overlapping nanoclusters on the cell surface. In addition, RAS isoform association with nanoclusters is dynamic and differentially mediated by GDP/GTP-bound state, lipid composition, HVR-interacting protein scaffolds and the actin cytoskeleton [42]. The mosaic of non-overlapping distributions is proposed to influence access to RAS regulators and effectors and mediate isoform-specific signalling.

KRAS nanoclustering is principally driven via electrostatic interactions with anionic lipids. Phosphatidic acid (PA) and phosphatidylserine (PS), but not phosphatidylinositol-4,5-bisphosphate (PIP2), are enriched in KRAS nanoclusters [43,44]. Depletion of cellular PS results in redistribution of KRAS from the plasma membrane [43]. The mechanism by which only a subset of anionic phospholipids was enriched was unclear, until recent work revealed that the polybasic domain of KRAS contains a code specifying lipid sorting. Sequential polybasic domain substitution of each of the six lysine residues to uncharged glutamine resulted in significant changes in co-localisation with PA, PS, PIP2 and PIP3 [45]. Changing the lysine residues to arginine, in order to retain the overall net charge of the polybasic domain, also changed the specificity for anionic lipids, indicating that charge is not the only determining factor. In addition to positioning of individual lysine residues influencing co-localisation with specific lipids, nanoclustering was also sensitive to the length and saturation of the phospholipid fatty acid chains [45]. Dynamic changes in HVR tertiary structure underlying these preferences were modelled and likely resemble a mechanism used by some other GTPases to fine-tune membrane interactions and function.

Another feature of RAS organisation on the membrane that has emerged recently is that activated RAS isoforms adopt a putative dimeric state. While biophysical studies using model membranes and fully processed KRAS4B failed to detect dimerisation [46], cell-based experiments provide clear evidence of KRAS4B dimerisation via the α4–α5 interface (Figure 2), suggesting an additional requirement for cellular factors or context [47]. A dimerisation-defective D154Q mutant failed to promote cell proliferation and exhibited impaired tumour initiation. The potential reason for this was the requirement for KRAS dimerisation to promote effective RAF heterodimerization and signalling. RAS dimerisation also provides a plausible mechanism for explaining the long-standing mystery of how wild-type RAS isoforms might antagonise oncogenic RAS mutant signalling [48,49]. For example, a wild-type-mutant RAS heterodimer might represent an ineffective combination for promoting RAF dimerisation. Since RAS dimerisation represents a therapeutic target, a synthetic protein inhibitor has been identified that inhibits HRAS and KRAS homo-dimerisation and signalling [50].

## RAS mutation-specific signalling

97% of Ras mutations occur at codons 12, 13 and 61, whereby single base substitutions can convert each codon into six different amino acids [3]. Although these mutations are all activating, they are not equally transforming [51–53]. Biochemical and structural analyses have revealed KRAS mutation-specific effects on nucleotide binding, GTP hydrolysis and effector interactions that predict significant differences in biological readouts [54,55]. For example, while G13D and Q61L exhibit rapid nucleotide exchange, the variable lengths of time that the other mutants are GTP-bound is dominated by differences in their relative GAP-mediated GTP hydrolysis rates. Codon 12, 13 and 61 mutations also variably specify decreases in affinity for their effector CRAF of up to 7-fold [54]. Although it is unclear if the decrease in RAF affinity will favour improved engagement of other effector pathways resulting in different mutations displaying different spectrums of effector activity.

Consistent with the notion that different mutations specify differences in RAS biology, cell-based studies have shown that different signalling outputs and phenotypic responses are associated with KRAS codon 12 versus codon 13 mutations [56–59]. For example, codon 12 mutant KRAS expression favoured anchorage-independent growth and resistance to apoptosis, whereas cells expressing codon 13 mutant KRAS died soon after reaching confluency [56]. Patient data also suggest that mutation-specific biology is relevant. Highly biased distributions of mutations are observed for each isoform with KRAS favouring codon 12, NRAS codon 61 and HRAS codons 12 and 61 (Figure 2) [3]. These distributions may reflect isoform-specific differences in mutagen exposure or sensitivity or differences in the oncogenic potential of each mutation. *In vivo* studies point to the different oncogenic potential of individual mutations. Human melanomas are associated with codon 61 mutants, and NRAS^Q61R^ is more efficient at promoting melanoma than NRAS^G12D^ in mice [60]. Similarly, another *in vivo* study showed that NRAS^Q61R/+^ mice mutants displayed higher leukaemogenic activity than NRAS^G12D/+^ [61].

Progress in this area has been limited by the availability of only a few genetically engineered mouse strains (Lox-Stop-Lox-(LSL-)HRAS^G12V/+^, LSL-KRAS^G12V/+^, LSL-KRAS^G12D/+^, LSL-NRAS^G12D/+^, LSL-NRAS^Q61R/+^) precluding comparisons of most RAS variants in a common model system. To overcome this, Winters et al. [62] developed a novel CRISPR library model to investigate a panel of KRAS oncogenic variants. Here, they used AAV/Cas9-mediated gene editing to simultaneously introduce 12 different KRAS mutations into each mouse and then used next-generation sequencing to measure the tumour burdens associated with each mutation. The study showed that the KRAS mutant variants have different oncogenic potential in lung and pancreas, but consistent with human patient data, G12V and G12D were consistently the most frequently observed mutations. This indicates that different RAS mutations that result in subtle differences in activity may have significant consequences on their resultant oncogenicity. This novel model has great potential to efficiently and quantitatively study relative RAS variant contributions to oncogenesis and responses to therapy.

## Concluding remarks

*In vitro*, *in vivo* and clinical approaches have contributed to a deep understanding of RAS biology and an appreciation of the evident complexity of the network biology and the heterogeneity of responses. Despite this, it remains difficult to pin down the answers to fundamental questions such as what are the relative contributions of the RAS isoforms to RAS biology, what makes KRAS more oncogenic than the other isoforms and how does wild-type RAS modulate oncogenic RAS signalling? While the answers are likely to be context-dependent, it is important that consensus is established around the selection of robust models and analysis pipelines that should be used in order to identify the most therapeutically tractable insights.

## Abbreviations

FTIs: farnesyltransferase inhibitors
GTP: guanosine triphosphate
HVR: hypervariable region
PA: phosphatidic acid
PIP2: phosphatidylinositol-4,5-bisphosphate
PS: phosphatidylserine
RTK: receptor tyrosine kinase
